# Shaping of the host cell by viral accessory proteins

**DOI:** 10.3389/fmicb.2015.00142

**Published:** 2015-02-23

**Authors:** Nadine Laguette, Monsef Benkirane

**Affiliations:** Molecular Virology Laboratory, Centre National de la Recherche Scientifique, Institute of Human Genetics CNRS UPR1142Montpellier, France

**Keywords:** HIV, accessory proteins, restriction factors, intrinsic immunity, sensing

Optimal viral replication relies on the ability of viruses to use cellular resources and to overcome the intracellular defense mechanisms. The human immunodeficiency virus (HIV) is no exception to the basic rules governing viral replication cycles. Indeed, to gain a foothold into host cells and disseminate, HIV needs to efficiently complete a series of steps, including entry, reverse transcription of its RNA genome into DNA competent for integration, and transcription of the integrated provirus into RNA molecules that will either be translated into progeny virion protein components, or constitute viral genomes. At each of these steps, cellular components, such as dNTPs, NTPs and amino acids, as well as cellular pathways are mobilized. Some of the latter passively serve the viral life cycle whereas some are actively high-jacked and side-tracked from their usual cellular function. While the presence of such resources and pathways within host cells facilitates viral replication, they do not suffice to render cells permissive to HIV infection. In fact, cells possess an arsenal of detection mechanisms (pathogen recognition receptors) and proteins that can block specific steps of the viral life cycle (restriction factors) that together constitute the intrinsic immune system.

Due to its limited genome size and the small number of encoded proteins, HIV-derived proteins, including accessory proteins (Vpr, Vpx, Vpu, Vif, and Nef), are highly pleiotropic and can contact numerous cellular partners. These proteins, which were initially coined as “accessories” because they appeared to be virtually unnecessary for *in vitro* viral replication in permissive cells, are encoded by lentiviral genomes in addition to major structural and enzymatic proteins (Gag, Pol, and Env) and regulatory proteins (Tat and Rev). The importance of accessory proteins can be evidenced as inefficient viral spread, delay in replication curves or low viral loads upon disruption of their corresponding open reading frames (ORF) in non-permissive cells and *in vivo*. Because these accessory proteins are key virulence factors, they have been the subject of intense investigation over the past decades. While this has allowed the identification of several cellular pathways and processes they subvert, the outcome of the established interactions remains sometimes elusive. Nonetheless, major functions of these accessory proteins may be grossly classified as: (i) counteraction of cellular restriction factors (ii) escape from innate immune sensing, (iii) disturbance of cellular pathways, and (iv) enhancement of viral infectivity. Importantly, no accessory protein has been shown to date to fit all four categories, but it is consensually admitted that more investigations are needed for full characterization of their function. This review collection aims at discussing recent advances in our understanding of manipulation of host cells by the HIV type 1 (HIV-1) and type 2 (HIV-2), with a particular focus on viral accessory proteins. Importantly, because our understanding of the function of viral accessory proteins frequently requires a better understanding of the antagonized cellular proteins or processes, these are also discussed.

To address these aspects, this review series starts by relating our current understanding of Nef function (Basmaciogullari and Pizzato, 2014), a master regulator of intracellular trafficking pathways. Particular emphasis is put on Nef-dependent enhancement of viral infectivity, an as of today poorly understood phenotype. Interestingly, together with Vpr, Nef is the only other HIV accessory protein for which a *bona fide* antagonized cellular restriction factor has not been identified to date. Similar to Nef, Vpr was documented to disturb several cellular pathways (Guenzel et al., 2014), with a major consequence being a potent cell cycle arrest. However, this major outcome was recently shown to be a mere side effect of Vpr-induced activation of a cellular SLX4 endonuclease complex (Bregnard et al., 2014; Laguette et al., 2014). Activation of the SLX4 complex likely serves to allow the processing of virion-derived reverse transcripts to favor escape from innate immune detection. This function is similar to that proposed for the TREX1 cellular exonuclease (Yan et al., 2010). Importantly, TREX1 and SLX4 complex activation are only two of the many ways by which the HIV virus evades innate immune sensing. While a particular focus is given to TREX1 (Hasan and Yan, 2014), this review also discusses the potential role played by the cyclic GMP-AMP Synthase (cGAS) pathogen recognition receptor (Gao et al., 2013).

To the contrary of Nef and Vpr, the other HIV-1 and HIV-2 accessory proteins, Vpu, Vif, and Vpx, have been shown to directly counteract cellular restriction factors. However, in recent years, a role for these restriction factors in detection of infections has also been described, suggesting that counteraction of these proteins serves in both alleviating the restriction and in escape from innate immune detection. Indeed, Vif, Vpu, and Vpx can directly target APOBEC3G, Tetherin and SAMHD1, respectively. Because of the dual role played by these restriction factors, it is both important to understand the molecular mechanism underlying the antagonism by accessory proteins and the function fulfilled by the cellular protein (Feng et al., 2014; Moris et al., 2014; Roy et al., 2014; Sauter, 2014; Schaller et al., 2014).

This review collection should therefore help the reader have an overview of the conflicting forces that underlie the interactions established by HIV with the host cell, and in particular of the complex relationship between viral accessory proteins and their cellular partners. This review series highlights how progress made in the understanding of the HIV life cycle has also impacted on our understanding of important cellular processes, especially with regards to the mechanisms underlying molecular aspects of the innate immune system. In fact, while the complexity of the interactions established between viral and cellular proteins precludes definite conclusions as of today, understanding their contribution to HIV-associated pathogenesis is likely to be the next big challenge in the field.

## Conflict of interest statement

The authors declare that the research was conducted in the absence of any commercial or financial relationships that could be construed as a potential conflict of interest.
